# Gemcitabine–Doxorubicin Combination Polymer-Drug Conjugate Prepared by SPAAC Click Chemistry: In Vitro Characterization

**DOI:** 10.3390/ijms26062798

**Published:** 2025-03-20

**Authors:** Omotola D. Gbadegesin, Simeon K. Adesina

**Affiliations:** Department of and Pharmaceutical Sciences, Howard University, Washington, DC 20059, USA

**Keywords:** strain-promoted [3 + 2] azide–alkyne cycloaddition, SPAAC, combination chemotherapy, ovarian cancer, gemcitabine, doxorubicin, polymer–drug conjugate

## Abstract

Combination chemotherapy is preferred for the treatment of ovarian cancer (OC). Systemic toxicity, however, frequently limits the effectiveness of treatment. Polymer–drug conjugates (PDCs) containing synergistic combinations of chemotherapeutic drugs can be used to enhance therapeutic efficacy. We earlier reported the use of a strain-promoted [3 + 2] azide–alkyne cycloaddition (SPAAC)-mediated polymerization method for the preparation of single-drug PDCs. In this report, the polymerization method was used to prepare gemcitabine–doxorubicin combination PDC. The PDC had a high molecular weight (M_w_ 1360 kDa) and high drug loading (36.6% weight gemcitabine; 7.0% weight doxorubicin). It demonstrated cathepsin B-catalyzed drug release at pH 5.0 and good hydrolytic stability at pH 7.4. The combination index analysis of free gemcitabine and free doxorubicin showed a concentration-dependent synergism (combination index < 1) in OVCAR-3 OC cells. Compared to individual gemcitabine PDC (the concentration that inhibited 50% growth (IC_50_) > 50 µg/mL) and doxorubicin PDC (IC_50_ = 1.79 µg/mL), the combination PDC (IC_50_ = 0.99 µg/mL) showed greater cytotoxicity against OVCAR-3 cells and was less cytotoxic than the equivalent free drug combination (IC_50_ = 0.11 µg/mL). The gemcitabine–doxorubicin combination PDC is promising for targeted combination chemotherapy of OC.

## 1. Introduction

Ovarian cancer (OC) has the highest fatality rate among all gynecological cancers globally [1,2,3]. Most patients have advanced OC at the time of diagnosis due to a lack of routine screening tests and non-specific symptoms that are difficult to identify from less significant abdominal symptoms, making treatment very challenging [1,3,4,5]. Combination chemotherapy is the mainstay for the treatment of advanced OC, with the first line being the 3-weekly 24 h cisplatin/paclitaxel intravenous co-infusion over six cycles [6,7]. The basis for combination chemotherapy is that multiple drugs with different mechanisms of action would serve as a multi-pronged approach to killing cancer cells and reducing the chances of the development of chemoresistance [8].

Gemcitabine and doxorubicin are FDA-approved for the treatment of advanced ovarian cancer. Gemcitabine inhibits DNA synthesis and induces cell cycle arrest in the synthesis phase [9,10]. Doxorubicin causes cytotoxicity by intercalating between DNA bases and altering the DNA structure. It also traps topoisomerase II in the double-strand cleaved form, introducing toxic double-strand breaks in the cell [11]. Based on their dissimilar mechanisms of action, doxorubicin and gemcitabine combination therapy has been tested in patients with OC and has shown synergistic effects with increased efficacy [12,13].

A key challenge in combination chemotherapy is systemic toxicity. Chemotherapeutic drugs are effective against cancer cells, but they also kill healthy cells, limiting the maximum dose that can be safely administered to an individual patient. Effective strategies are thus needed to maximize the therapeutic effects and minimize the non-specific toxicity of existing OC chemotherapeutic agents. Nano-chemotherapeutics, such as (Doxil^®^) and (Abraxane^®^), are used clinically for the treatment of advanced or recurrent OC. These have shown significant benefits over conventional chemotherapeutic agents. For instance, Doxil^®^ is a poly (ethylene glycol)-modified liposomal doxorubicin with longer blood circulation time, tumor accumulation, and reduced toxicity, compared to doxorubicin [14]. Abraxane^®^, a paclitaxel-loaded albumin-bound nanoparticle, solubilizes paclitaxel without Cremophor EL, the solubilizing agent in the standard paclitaxel formulation that is linked with paclitaxel-associated hypersensitivity reactions [15,16]. The carrier systems of these nanoparticle delivery systems encapsulate chemotherapeutic drugs, which are susceptible to ‘burst release’, the rapid and substantial release of drugs shortly after administration before reaching the target location [17,18]. This may result in therapeutic inefficacy and off-target toxicity in healthy cells.

Targeted drug conjugate systems, such as antibody–drug conjugates, peptide–drug conjugates, and polymer–drug conjugates, utilize the ‘prodrug approach’ to maximize therapeutic effects and minimize off-target toxicity. The active chemotherapeutic drugs are prepared as conjugates that are inactive while traversing the body until activated by specific stimuli in the tumor tissues. We have provided an extensive review of targeted drug conjugate systems for the chemotherapy of OC [19], and the reader is referred to it. This study is focused on polymer–drug conjugates (PDCs), the drug delivery systems where the active drugs are covalently attached to polymeric chains through stimuli-sensitive linkers. PDCs can be used to modify the biodistribution of small-molecule anticancer agents to prevent undesired distribution to off-target cells, thereby minimizing adverse side effects [20]. In addition, due to the leaky vasculature and impaired lymphatic drainage system present in tumor tissues, nanosized PDCs can preferentially accumulate in cancer tissues compared with healthy tissues. This phenomenon is called the enhanced permeability and retention (EPR) effect and is the basis for the passive targeting of cancer [21,22,23].

In our previous report, strain-promoted [3 + 2] azide–alkyne cycloaddition (SPAAC)-mediated step-growth polymerization was used to prepare individual gemcitabine and doxorubicin PDCs with high drug loading and high molecular weights [24]. We reported that the advantages of our strategy compared to other reported methods for the synthesis of PDCs for anticancer drug delivery “include modularity, absence of an initiator or catalyst, rapid progression under mild conditions, the ease of synthesis of the monomers, being amenable to different spacers and drugs, and the use of highly specific copper-free click chemistry” [24]. We present here a gemcitabine–doxorubicin combination PDC synthesized by a modification of the reported method. We hypothesized that the dual delivery of both drugs on a single polymer backbone may result in a greater anticancer effect than single-drug PDCs. To compare efficacy, the cytotoxic effects of the individual and combination PDCs were evaluated in vitro in OVCAR-3 human OC cell line using the CellTiter-Glo^®^ 2.0 assay, and were compared with the free drugs at equivalent concentrations.

## 2. Results and Discussion

### 2.1. Synthesis and Characterization of P-Gem/Dox

**Scheme 1:**
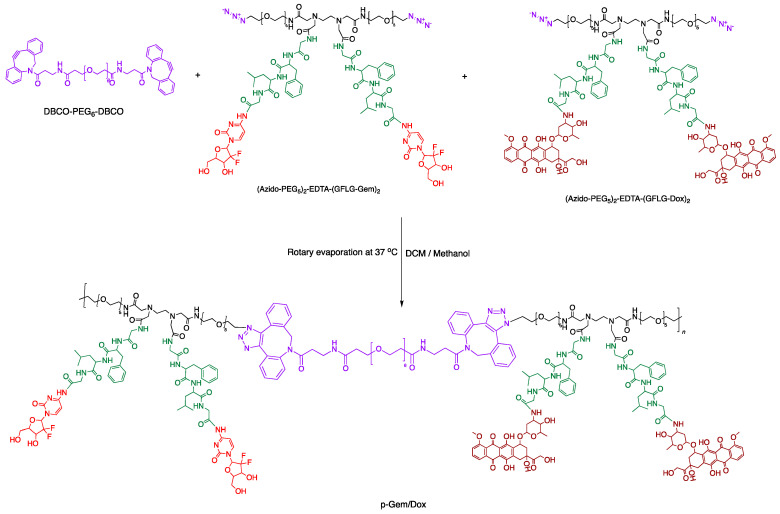
Synthesis of gemcitabine–doxorubicin-combination PDC (p-Gem/Dox) using SPAAC reaction between azide-terminated homo-bifunctional (azidoPEG_5_)_2_-EDTA-(GFLG-Dox)_2_ and (azidoPEG_5_)_2_-EDTA-(GFLG-Gem)_2_ drug-coupled monomers, and DBCO-PEG_6_-DBCO, a dibenzoazacyclooctyne-terminated homo-bifunctional PEG monomer.

**Figure 1:**
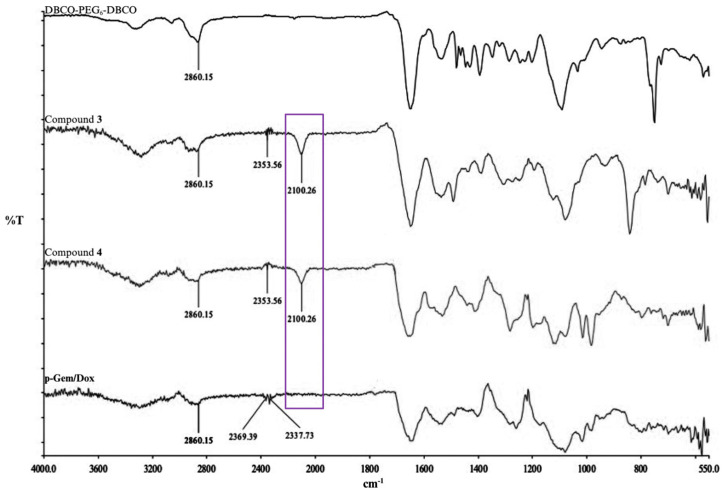
Overlaid FT-IR spectra of DBCO-PEG_6_-DBCO, (azidoPEG_5_)_2_-EDTA-(GFLG-Gem)_2_ (**3**), (azidoPEG_5_)_2_-EDTA-(GFLG-Dox)_2_ (**4**), and p-Gem/Dox. The absence of the azide stretch at around 2100 cm^−1^ showed that the azide-terminated starting monomers had been used up.

We previously employed SPAAC-mediated step-growth polymerization for the synthesis of the single PDCs (p-Gem and p-Dox), containing gemcitabine and doxorubicin, respectively, as a proof of concept and to validate the novel approach [24]. In this present work, we report the utilization of SPAAC-mediated step-growth polymerization for combination drug delivery. The homo-bifunctional monomers, DBCO-PEG_6_-DBCO, (azidoPEG_5_)_2_-EDTA-(GFLG-Gem)_2_, and (azidoPEG_5_)_2_-EDTA-(GFLG-Dox)_2_, were co-polymerized in a 1:1:1 stoichiometric ratio, respectively, to obtain gemcitabine–doxorubicin combination PDC (p-Gem/Dox) (Scheme 1). A DBCO-PEG_6_-DBCO solution was added in aliquots to the mixture of (azidoPEG_5_)_2_-EDTA-(GFLG-Gem)_2_ and (azidoPEG_5_)_2_-EDTA-(GFLG-Dox)_2_. Following the addition of each aliquot, the solvent was removed by rotary evaporation. This increased the concentrations of the reactants and caused the polymerization to occur rapidly and more efficiently [24,25]. Similarly to the single PDCs earlier reported, FT-IR analysis of p-Gem/Dox showed that the azide stretching frequency at around 2100 cm^−1^ in the azide-terminated homo-bifunctional (azidoPEG_5_)_2_-EDTA-(GFLG-Dox)_2_ and (azidoPEG_5_)_2_-EDTA-(GFLG-Gem)_2_ drug-coupled monomers was absent in p-Gem/Dox (Figure 1). This confirms that all the azide-terminated drug-coupled monomer molecules had been used up in the formation of higher molecular weight molecules [21]. This validates the versatility of our approach in the design of PDCs.

**Figure 2:**
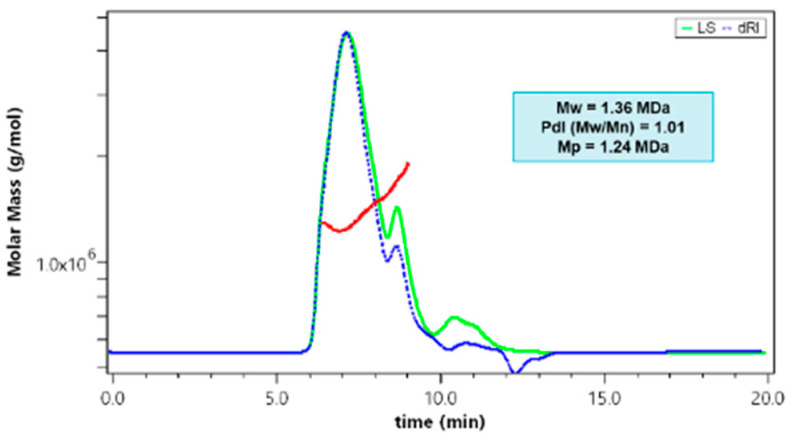
The SEC chromatogram of p-Gem/Dox showing the light scattering (LS, green line), differential refractive index (dRI, blue line), and molecular weight distribution (red).

The molecular weight of the synthesized p-Gem/Dox was characterized using SEC coupled with MALS and RI detectors (SEC/MALS-RI). The molecular weight distribution of p-Gem/Dox across the eluted peaks after SEC separation was measured (Figure 2) and the reported molecular weights are summarized in Table 1. We observed that the molecular weights obtained for the doxorubicin-containing PDCs (p-Gem/Dox and p-Dox) were greater than 1000 kDa while the PDC that contains gemcitabine alone (p-Gem) had molecular weights that were up to 40 kDa. The reason for this is not clear, but we believe that it has to do with differences in the drug-coupled monomers that were used in the SPAAC-mediated polymerization reactions. The methods of synthesis of the single-drug PDCs (p-Gem and p-Dox) and the dual-drug PDC (p-Gem/Dox) were different only in terms of the azide-terminated homo-bifunctional drug-coupled monomers that were used. For p-Gem and p-Dox, DBCO-PEG_6_-DBCO was reacted with (azidoPEG_5_)_2_-EDTA-(GFLG-Gem)_2_ and (azidoPEG_5_)_2_-EDTA-(GFLG-Dox)_2_, respectively, in a 1:1 stoichiometric ratio. While for p-Gem/Dox, DBCO-PEG_6_-DBCO was reacted with a mixture of (azidoPEG_5_)_2_-EDTA-(GFLG-Gem)_2_ and (azidoPEG_5_)_2_-EDTA-(GFLG-Dox)_2_ in a 1:1:1 stoichiometric ratio.

Compared to polymers formed by traditional step-growth polymerization methods, which are typically characterized by the presence of intermediate-sized molecules [26,27], the drug-coupled polymers in this study have high molecular weights with relatively narrow molecular weight distributions. The polydispersity index (PdI) of p-Gem/Dox based on its measured molecular weight distribution (Figure 2) was 1.01, indicating a narrow molecular weight distribution. In addition, the measured molecular weight of its most abundant species based on its RI signal (M_p_) was close to the measured weight-average molecular weight (M_w_) and number-average molecular weight (M_n_), indicating a relatively narrow molecular weight distribution similar to the previously reported single PDCs (Table 1).

In p-Gem/Dox, gemcitabine loading (36.6%) was greater than doxorubicin loading (7%), which was similar to the corresponding single drug-coupled polymers (Table 1). This could be due to the smaller molecular weight of gemcitabine (263 Da) compared with doxorubicin (543 Da). The larger size of doxorubicin and its anthraquinone ring may introduce steric hindrance leading to reduced efficiency of doxorubicin coupling to the polymer backbone. Compared with other reported studies, the prepared p-Gem/Dox using our SPAAC approach, had better drug loading. For instance, a poly (N-(2-hydroxypropyl) methacrylamide) (HPMA) copolymer bound to gemcitabine and doxorubicin via GFLG had 6.4% weight gemcitabine and 5.7% weight doxorubicin [28]. Another reported gemcitabine–doxorubicin combination PDC with hyaluronic acid as the polymer backbone had a ≤3.6% loading and a ≤5.0% loading for doxorubicin and gemcitabine, respectively [29].

### 2.2. In Vitro Cathepsin B-Catalyzed Cleavage of P-Gem/Dox

**Figure 3:**
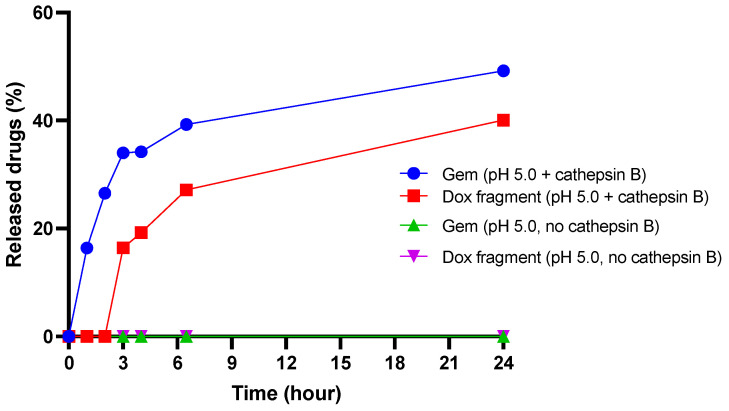
Cathepsin B-catalyzed release of gemcitabine and a doxorubicin fragment from p-Gem/Dox in the presence (blue and red lines, respectively) and absence (green and purple lines, respectively) of exogenous cathepsin B at pH 5.0. The percentages of the drugs released at the different time points were calculated from the peaks of the released gemcitabine and doxorubicin fragments versus the polymer-bound drug monitored by a diode array detector at 275 nm [30,31,32].

The cathepsin B-catalyzed cleavage of p-Gem/Dox was carried out to evaluate the release rate of gemcitabine and doxorubicin from the polymer conjugate. The release rate of gemcitabine from the polymer following enzyme cleavage of p-Gem/Dox showed a rapid initial release within 2 h, followed by a gradual release over the 24 h evaluation period (Figure 3). The RP-HPLC chromatogram for p-Gem/Dox cleavage test solution at pH 5 showed the release of gemcitabine (peak retention time 2.8 min) from p-Gem/Dox (peak retention time 8.0 min) in the presence of cathepsin B. No gemcitabine was released in the control experiment without cathepsin B (Supplementary Material, Figure S5).

With the evaluation of the doxorubicin release rate, no free doxorubicin was detected at the characteristic doxorubicin wavelength of absorption (480 nm) under the conditions of our study. A similar release pattern was observed for the individual PDCs of gemcitabine and doxorubicin in our earlier report [24]. A new peak (retention time 3.8 min) that was absent in the control without cathepsin B, was observed in the test at 3 h with detection at 275 nm (Supplementary Material, Figure S5). The release of this compound, which started appearing at 3 h in the test, also increased gradually when monitored at 275 nm over 24 h. This was similar to our observation with p-Dox, which released a doxorubicin fragment (retention time 3.8 min) when monitored at 275 nm [24].

The structure of doxorubicin consists of a hydrophobic anthraquinone ring and a hydrophilic amino sugar moiety, indicating that doxorubicin is amphiphilic [33]. The peak at a retention time of 3.8 min was attributed to the hydrophilic amino sugar moiety of doxorubicin, which does not absorb at 480 nm. As reported in our earlier paper, at the end of the cleavage experiment, a sample of the conjugate solution in the cleavage buffer was dissolved in acetonitrile and was analyzed by mass spectrometry [24]. The mass analysis showed masses (*m*/*z*) corresponding to free gemcitabine (calculated 264.1 [M + H]^+^, found 263.0), an adduct ion of the hydrophilic doxorubicin amino sugar moiety (calculated 179.0 [M + CH_3_OH + H]^+^, found 179.0), and a doxorubicin aglycone fragmentation product (calculated 482 [M + H]^+^, found 481.3) [34] (Supplementary Material, Figure S6).

The slower release of doxorubicin compared with gemcitabine may be due to hydrophobic interactions between doxorubicin molecules, hindering the enzyme’s access to GFLG and, hence, the reduced rate of enzyme-catalyzed cleavage [8]. In addition, the drug release pattern from the p-Gem/Dox in this study correlated with the result obtained from a similar p-Gem/Dox that was formed using an HPMA copolymer [28]. The researchers attributed the faster release of gemcitabine compared to doxorubicin to the small size of gemcitabine which causes less steric hindrance [28].

### 2.3. Hydrolytic Stability of P-Gem/Dox

**Figure 4:**
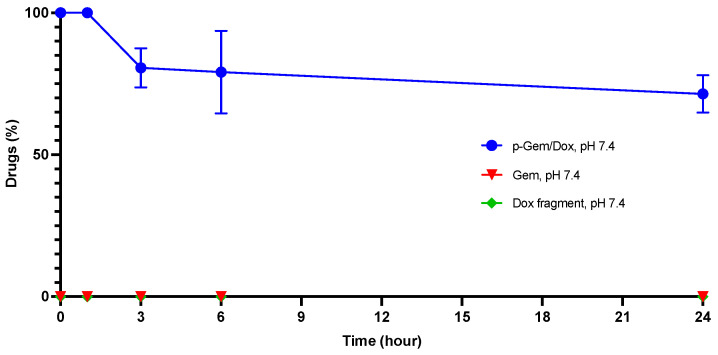
The hydrolytic release of gemcitabine and doxorubicin from p-Gem/Dox in PBS (pH 7.4). The decrease in the peak corresponding to polymer-bound gemcitabine–doxorubicin was monitored by a diode-array detector at 480 nm and the released drugs were monitored at 275 nm (n = 3) [31].

The in vitro stability of p-Gem/Dox was carried out in phosphate-buffered saline (PBS) (pH 7.4) to evaluate its aqueous stability at physiological pH. p-Gem/Dox demonstrated good stability in PBS (pH 7.4) (Figure 4), indicating that it could maintain good stability at physiological pH with efficient drug release in the tumor when cleaved by cathepsin B. The stability of p-Gem/Dox at pH 7.4 may be attributed to the high stability of GFLG and that of the amide bond which was used for the conjugation of the drugs to the polymer backbone. GFLG is more specific for cathepsin B than cathepsin B dipeptide substrates like Val-Ala or Val-Cit, and it is stable in plasma [35,36,37]. In addition, amide bonds have the propensity to form resonant structures, which confer great stability to amide bonds [38].

Although we observed some hydrolytic degradation of p-Gem/Dox at pH 7.4, we did not detect free drugs because no exogenous cathepsin B was present to cleave the GFLG linker. However, the slow release of a sugar fragment from doxorubicin under the conditions of the study may have contributed to the change in PDC area at the retention time of the PDC when analyzed by HPLC under the conditions for p-Gem/Dox detection.

### 2.4. In Vitro Cytotoxicity Evaluation

The cytotoxicity of the single-drug PDCs (p-Dox and p-Gem), the p-Dox and p-Gem admixture (50:50), and the p-Gem/Dox (dual-drug PDC) to OVCAR-3 OC cells was evaluated. The cell line is known to express cathepsin B [39]. Also, the most prevalent and aggressive histotype of OC, high-grade serous OC, is well represented by the OVCAR-3 cell line [40]. The cells were exposed to solutions of the PDCs (0.005, 0.05, 0.5, 5, and 50 μg/mL PDC equivalents of gemcitabine and doxorubicin) for 72 h. Gemcitabine HCl (GEM) and doxorubicin HCl (DOX) were used to evaluate the cytotoxicity of the free drugs. The CellTiter-Glo^®^ 2.0 assay was used for determining the percentage of viability of OVCAR-3 cells at 72 h post treatment. The assay gives a valid indication of cell viability by quantifying the amount of intracellular adenosine triphosphate as luminescent signals generated by metabolically active cells present in the culture [41].

#### 2.4.1. Cytotoxicity Evaluation of p-Dox

**Figure 5:**
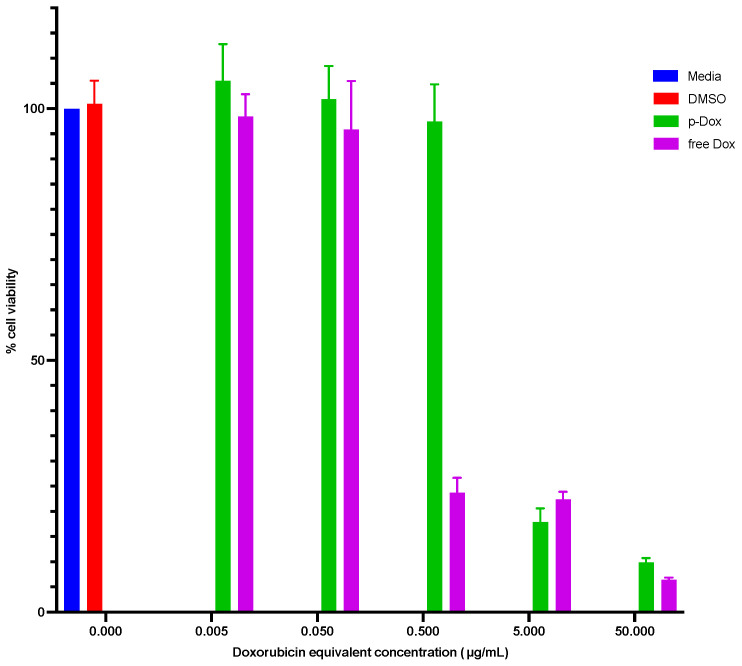
Percentage of viability of OVCAR-3 cells at 72 h post treatment with p-Dox and DOX at different concentrations. Controls represent cell culture media and DMSO in media. Data represent mean ± SD (n = 4).

Doxorubicin HCl (DOX) is approved for the treatment of metastatic OC [42]. For a direct comparison with DOX, the amount of p-Dox containing the same amount of DOX as DOX in solution was used. The percentage of viability of OVCAR-3 cells that were treated with p-Dox and DOX relative to untreated controls was determined. The results showed a concentration-dependent inhibition of cell growth, with the free drug being more cytotoxic than the polymer-bound drug (Figure 5). The concentration that inhibited the growth of 50% of the cells (IC_50_ value) of p-Dox (1.79 ± 0.17 μg/mL) was about ten times higher than the IC_50_ value of DOX (0.17 ± 0.03 μg/mL). Compared with small molecule drugs, which diffuse freely into the cells, PDCs may enter the cells by micropinocytosis which is a slower process than diffusion [43,44]. To match the cytotoxicity of the small molecule DOX, effective cell internalization followed by cathepsin B-catalyzed drug release is required for p-Dox [32].

Literature reports have shown that GFLG–doxorubicin-coupled polymers inhibit the proliferation of OC cells even though the in vitro release of doxorubicin from polymers was very slow, and no active targeting to cell surface receptors was employed [43,45,46,47,48]. Malugin et al. demonstrated that the liberation of doxorubicin from an HPMA copolymer–doxorubicin conjugate occurred after cell internalization and cleavage of GFLG by lysosomal cathepsin B [46]. They concluded that as long as the study conditions allow for endocytosis and the lysosomal degradation of PDCs, the HPMA copolymer–doxorubicin conjugate can induce cancer cell death [46]. A similar conclusion can also be made for the observed cytotoxicity of p-Dox because cathepsin B-expressing OC cells (OVCAR-3) were used in this study [39]. Cathepsin B secreted into the media by the cells could also be responsible for the drug release from the conjugate. The released drug would then be able to freely diffuse into the cells to induce cell death. In addition, the cells could also die by necrosis due to the toxic effect of p-Dox on the cell membrane [43,45].

#### 2.4.2. Cytotoxicity Evaluation of p-Gem

**Figure 6:**
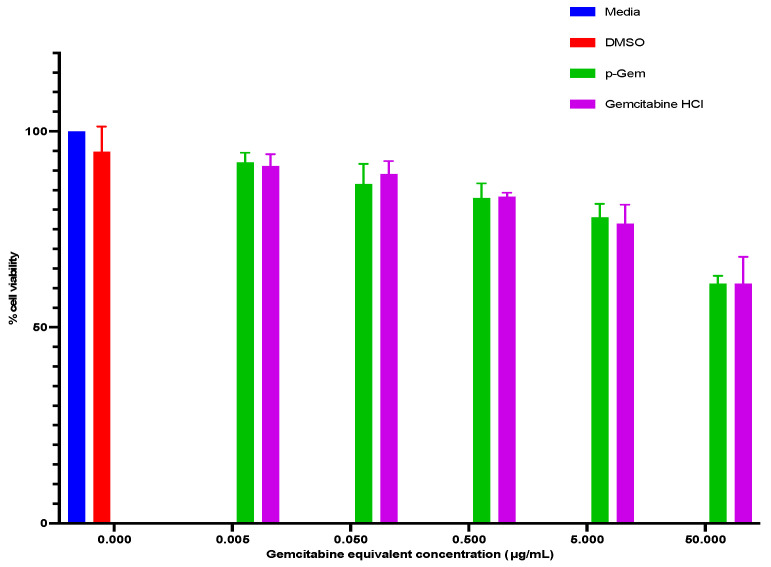
Percentage of viability of OVCAR-3 cells at 72 h post treatment with p-Gem and GEM at different concentrations. Controls include cell culture media and DMSO in media. Data represent mean ± SD (n = 4).

Gemcitabine HCl (GEM), used with carboplatin, is approved for the treatment of advanced OC that is resistant to first-line chemotherapy [42]. The percentage of viability of OVCAR-3 cells that were treated with solutions of p-Gem and GEM, relative to untreated control cells, was determined. Our data show no difference in the inhibition of proliferation of cells between cells treated with the polymer-bound gemcitabine compared to free gemcitabine (Figure 6). This observation can be explained based on the release pattern of gemcitabine from p-Gem. We reported earlier that the cleavage of p-Gem by cathepsin B was very fast [24]. Within 72 h, almost all the conjugated gemcitabine would have been released due to the rapid cathepsin B-catalyzed cleavage of p-Gem. Since an equivalent drug concentration based on gemcitabine content in the p-Gem was used, at 72 h post treatment, it is expected that the cells would be exposed to similar gemcitabine concentrations in solution after PDC cleavage and gemcitabine release by cathepsin B. Compared with p-Dox, however, both p-Gem and GEM were less potent against OVCAR-3 cells at the tested concentrations, and thus, had large IC_50_ values (>50 µg/mL) (Table 2).

#### 2.4.3. Cytotoxicity Evaluation of the Combination PDCs

**Figure 7:**
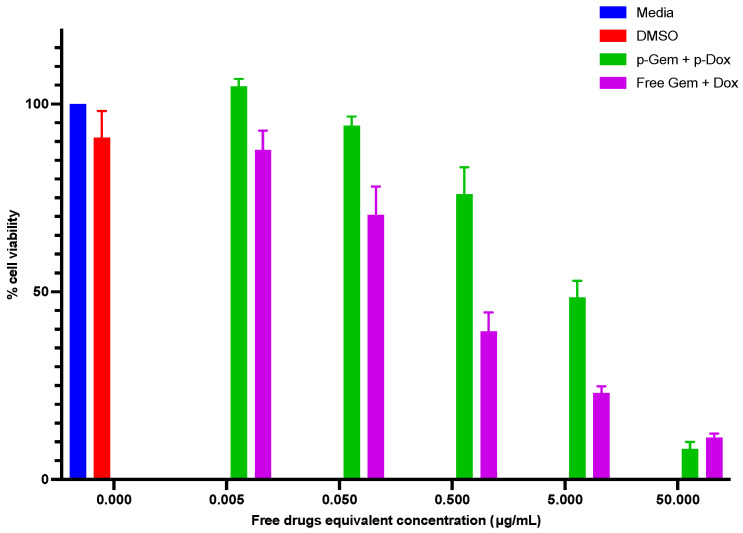
Percentage of viability of OVCAR-3 cells at 72 h post treatment with p-Dox + p-Gem admixture (50:50) and GEM + DOX admixture (50:50) at different concentrations. Controls represent cell culture media and DMSO in media. Data represent mean ± SD (n = 4).

The percentage of viability of OVCAR-3 cells 72 h after treatment with the 50:50 admixtures of p-Dox + p-Gem and GEM + DOX, relative to untreated control cells, were determined and compared. Growth inhibition in the cells increased with increasing concentrations of the treatments (Figure 7). The 50:50 admixture of p-Dox + p-Gem (IC_50_ value = 3.87 ± 1.22 μg/mL) was less cytotoxic to the cells than the 50:50 admixture of GEM + DOX (IC_50_ value = 0.24 ± 0.12 μg/mL). This higher cytotoxicity of the free drug combination when compared to the combination of p-Dox + p-Gem could be due to the ability of the free drugs to enter into the cells more freely and rapidly compared to the PDCs. In addition, conjugated drugs are not expected to be active until release is triggered by the cathepsin B cleavage of GFLG.

A higher cytotoxicity was observed when the cells were treated with p-Gem and p-Dox as a 50:50 admixture (IC_50_ value = 3.87 ± 1.22 µg/mL) compared to p-Gem alone (IC_50_ value > 50 µg/mL). This observation can be explained by the addition of doxorubicin, which is more potent than gemcitabine. Gemcitabine is a deoxycytidine analog that is converted to its active metabolites, gemcitabine diphosphate, and gemcitabine triphosphate, by deoxycytidine kinase [49]. Gemcitabine diphosphate irreversibly inhibits ribonucleotide reductase, which produces the deoxyribonucleotides (including deoxycytidine triphosphate) necessary for DNA synthesis, and gemcitabine triphosphate competes with deoxycytidine triphosphate for incorporation into an elongating DNA chain. The treatment of cells with gemcitabine rapidly depletes deoxycytidine triphosphate pools, inhibiting DNA synthesis and inducing cell cycle arrest in the synthesis phase [10]. However, the ‘arrested’ cells can recover without further treatment [9,10]. We have shown earlier that the cleavage of p-Gem by cathepsin B was faster than p-Dox [24]. In this study, the release of gemcitabine from p-Gem/Dox was also faster than doxorubicin. Hence, the fast release of gemcitabine from p-Gem in the p-Dox + p-Gem admixture (50:50) would likely arrest the cells in the DNA synthesis phase, and the release of doxorubicin later will exploit the vulnerable state of the cells before they adequately recover.

**Figure 8:**
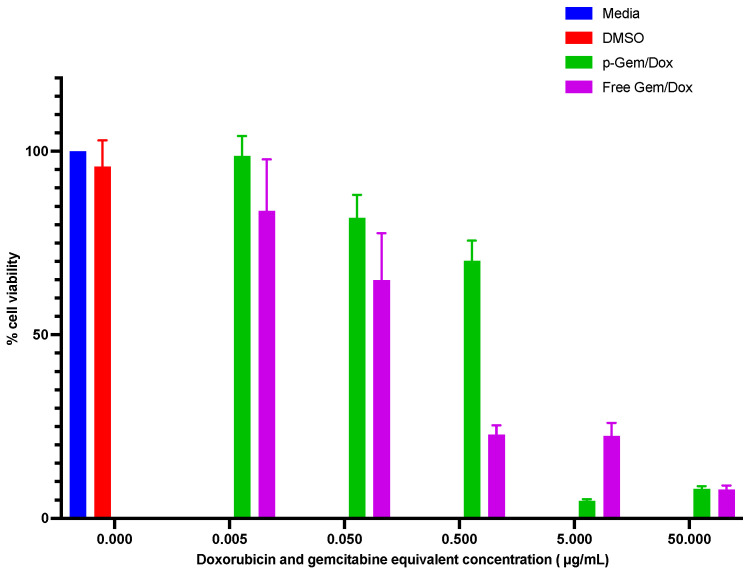
Percentage of viability of OVCAR-3 cells at 72 h post treatment with p-Gem/Dox and GEM + DOX admixture (36.6:7) at different concentrations. Controls represent cell culture media and DMSO in media. Data represent mean ± SD (n = 4).

Additionally, the percentage of viability of OVCAR-3 cells that were treated with solutions of p-Gem/Dox (where gemcitabine and doxorubicin were coupled to the same polymer backbone) and a GEM + DOX admixture (36.6:7), relative to untreated control cells, were also determined. A GEM + DOX admixture (36.6:7) was used as the free drug control for p-Gem/Dox based on the drug content in p-Gem/Dox (36.6% weight gemcitabine and 7% weight doxorubicin). Growth inhibition in the cells was also concentration-dependent (Figure 8), and p-Gem/Dox (IC_50_ value = 0.99 ± 0.06 µg/mL), being a conjugate, was less cytotoxic to the cells compared to the free drug admixture (IC_50_ value = 0.11 ± 0.04 µg/mL). Interestingly, the IC_50_ value of p-Gem/Dox was about four times lower than the IC_50_ value of the 50:50 p-Dox + p-Gem admixture (3.87 ± 1.22 µg/mL). This indicates that the conjugation of doxorubicin and gemcitabine to the same polymer backbone confers an increase in potency against OVCAR-3 cells, compared to the physical mixture of the individual conjugates of each drug (i.e., 50:50 p-Dox + p-Gem admixture). This can be explained by the ability of a polymer conjugate bearing two different drugs on the same polymer backbone to share the same biodistribution and enzyme cleavage kinetics, compared to the individual conjugates which may exhibit distinct kinetics. In addition, our result is similar to what was reported by Vogus et al. who reported that the combination conjugate of gemcitabine and doxorubicin was more toxic than the physical mixture of the individual drug conjugates in triple-negative breast cancer cells [29].

#### 2.4.4. Combination Index Analysis

Combination chemotherapy has demonstrated efficacy in the treatment of OC, with the current first-line chemotherapy being platinum—taxane combination therapy [6]. When developing a new combination therapy, it is important to determine the overall effect of such a drug combination, which may be an additive effect, synergism, or antagonism. The method developed by Chou and Talalay is widely utilized for evaluating the effect or interaction of combination treatments [50]. The Combination Index (CI) is determined through the assessment of the effects of individual drugs as well as their combined effects. “CI values = 1, <1, and >1 are regarded as an additive effect, synergism, and antagonism, respectively” [50].

**Figure 9:**
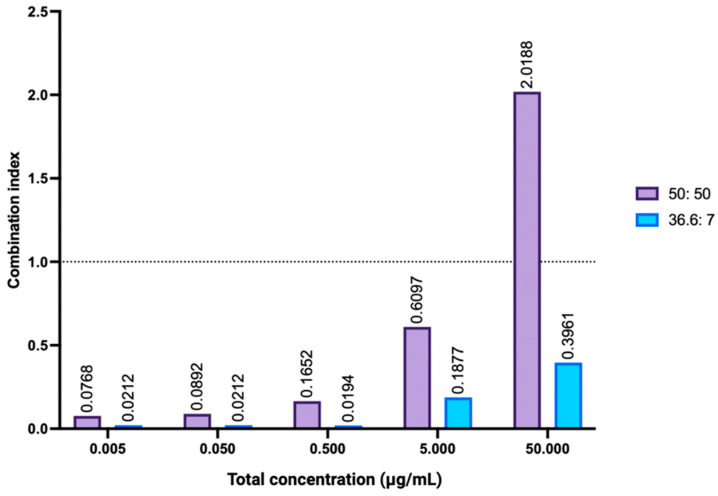
Combination index (CI) analysis of GEM/DOX combinations at ratios of 50:50 and 36.6:7, respectively, for GEM and DOX. Percentages of viability of OVCAR-3 cells at 72 h post treatment with GEM + DOX admixture (50:50) and GEM + DOX admixture (36.6:7) were analyzed using the COMPUSYN software (Version 1.0) to determine the CI values.

The percentage of viability of OVCAR-3 cells at 72 h post treatment with the GEM + DOX admixture (50:50) and the GEM + DOX admixture (36.6:7) were analyzed using the COMPUSYN software (Version 1.0) to determine the CI values. The effects of free drug combinations at ratios of 50:50 and 36.6:7, respectively, for GEM and DOX, were analyzed and compared. For the GEM + DOX admixture (36.6:7), the effect was synergistic (CI values < 1), and the synergism decreased with increasing drug concentrations (Figure 9). This was the same for the GEM + DOX admixture (50:50), except at the highest concentration (50 µg/mL), where the effect was antagonistic. The antagonism at a 50 µg/mL concentration can be explained by the IC_50_ values obtained for GEM (>50 µg/mL) and DOX (0.17 ± 0.03 µg/mL). The effect of doxorubicin alone had probably overshadowed the effect of gemcitabine, such that the effect of gemcitabine in the GEM + DOX admixture (50:50) at such a high concentration would be insignificant. Based on this analysis, GEM/DOX combinations at high equivalent concentrations may not produce more desired effects.

The CI values for the GEM + DOX admixture (36.6:7) and the GEM + DOX admixture (50:50) at 50% relative viability (EC_50_) were also compared. At EC_50_, the GEM + DOX admixture (36.6:7) showed very strong synergism (CI value = 0.02731) while the GEM + DOX admixture (50:50) showed strong synergism (CI value = 0.19325) [50]. This analysis supported the higher potency of p-Gem/Dox (the dual-drug PDC containing 36.6% weight of gemcitabine and 7% weight of doxorubicin) against OVCAR-3 cells compared to the p-Dox + p-Gem admixture (50:50) as discussed in Section 2.4.3. Synergistic effects typically arise when chemotherapeutic agents work through distinct mechanisms of action or when the impact of one agent enhances the sensitivity of cells to another agent’s effects [8]. Our data support the combination of both drugs for the treatment of OC.

## 3. Materials and Method

### 3.1. Materials

The dimethyl sulfoxide (DMSO), dichloromethane (DCM), methanol, N, N′-dimethylformamide (DMF), lithium chloride (LiCl), sodium phosphate monobasic monohydrate, EDTA disodium salt dihydrate, sodium hydroxide pellets, and 1,4-dithiothreitol (DTT) were obtained from Sigma-Aldrich (Burlington, MA, USA). Dibenzoazacyclooctyne-hexa (ethylene glycol)-dibenzoazacyclooctyne (DBCO-PEG_6_-DBCO) was obtained from AxisPharm (San Diego, CA, USA). The polystyrene standard (PS80317) was obtained from Pressure Chemical Co. (Pittsburgh, PA, USA). The purified native human cathepsin B was obtained from Athens Research and Technology (Athens, GA, USA). The human ovarian carcinoma cell line (NIH: OVCAR-3, ATCC^®^ HTB-161) was obtained from the American Type Culture Collection (ATCC) (Manassas, VA, USA). The RPMI-1640 medium was purchased from Invitrogen (Carlsbad, CA, USA). The fetal bovine serum was obtained from Fisher Scientific (Pittsburgh, PA, USA). The penicillin/streptomycin was obtained from Cellgro (Manassas, VA, USA). The CellTiter-Glo^®^ 2.0 Cell Viability Assay was obtained from Promega (Madison, WI, USA). The gemcitabine hydrochloride (HCl) and doxorubicin HCl were obtained from Biosynth Limited (Compton, UK). All materials were used as received.

### 3.2. Synthesis of Gemcitabine–Doxorubicin Combination PDC (P-Gem/Dox)

Two azide-terminated homo-bifunctional drug-coupled monomers, (azidoPEG_5_)_2_-EDTA-(GFLG-Gem)_2_ and (azidoPEG_5_)_2_-EDTA-(GFLG-Dox)_2_, were synthesized as previously reported [24]. Equimolar concentrations of the two homo-bifunctional drug-coupled monomers and DBCO-PEG_6_-DBCO, a dibenzoazacyclooctyne-terminated homo-bifunctional monomer, were reacted via SPAAC-mediated step-growth polymerization to prepare p-Gem/Dox, a combination PDC bearing both doxorubicin and gemcitabine on the same polymer backbone. Briefly, separate solutions of (azidoPEG_5_)_2_-EDTA-(GFLG-Gem)_2_ (0.02514 g; 29.8 mM in 400 μL of methanol), (azidoPEG_5_)_2_-EDTA-(GFLG-Dox)_2_ (0.03182 g; 29.8 mM in 400 μL of methanol), and DBCO-PEG_6_-DBCO (0.02144 g; 29.8 mM in 800 μL of DCM) were prepared. The prepared solutions of (azidoPEG_5_)_2_-EDTA-(GFLG-Dox)_2_ and (azidoPEG_5_)_2_-EDTA-(GFLG-Gem)_2_ were combined in a glass scintillation vial, and vortexed. DBCO-PEG_6_-DBCO solution (0.7 equivalent; 560 μL) was added to the mixture of the monomers and vortexed before removing the solvent mixture by rotary evaporation (Rotavapor R-300, Buchi, Switzerland, 37 °C, 60 rpm). The remaining 0.3 equivalent of the DBCO-PEG_6_-DBCO solution was added in two equal portions (120 μL each time) to the diluted solution of the product (1600 μL of a DCM–methanol 50/50 solvent mixture was used each time) followed by the removal of the solvent by rotary evaporation. The addition of the solvent and the rotary evaporation were repeated ten times to allow for a complete reaction. The PDC product was analyzed by Fourier-transform infrared (FT-IR) spectroscopy (Perkin Elmer Spectrum 100 FT-IR spectrometer, Perkin Elmer, Waltham, MA, USA) to monitor the azide functional groups of the azide-terminated monomers.

### 3.3. Molecular Weight Characterization

The molecular weight of p-Gem/Dox was characterized by size-exclusion chromatography (SEC) using a high-performance liquid chromatography (HPLC) system (Agilent, Palo Alto, CA, USA) equipped with the TSKgel Guard Alpha (6 mm ID × 4 cm, 13 μm) and TSKgel Alpha-3000 (7.8 mm ID × 30 cm, 7 μm) columns (Tosoh Bioscience LLC, King of Prussia, PA, USA). DAWN HELEOS-II multi-angle laser light scattering (MALS) and Optilab T-rEX refractive index (RI) detectors (Wyatt Technology, Santa Barbara, CA, USA) were employed, and DMF with 10 mM LiCl (1 mL/min flow rate) was used as the isocratic mobile phase. A sample solution of p-Gem/Dox (20 mg/mL) was prepared in DMF with 10 mM LiCl, and 100 μL was injected into the chromatographic system. RI change was measured differentially at a 660 nm laser wavelength using the Optilab T-rEX RI detector, and ASTRA software (v6.1.2.84, Wyatt Technology) was used to calculate the specific RI value of p-Gem/Dox (Supplementary Material, Figure S1). Polystyrene standard (PS 80317, 30 kDa) prepared at 4 mg/mL in DMF with 10 mM LiCl was used for MALS normalization.

### 3.4. Drug Content Evaluation

Gemcitabine and doxorubicin contents in p-Gem/Dox were determined by ultraviolet–visible (UV–vis) and fluorescence spectroscopy, respectively, on a Horiba Duetta spectrometer (HORIBA Scientific, Piscataway, NJ, USA). Triplicate solutions of p-Gem/Dox (100 μg/mL), prepared using 90% *v*/*v* DMF in water, were used for each analysis. The gemcitabine absorption spectra were collected from 200 to 400 nm at a 2 nm step increment, a 0.05 s integration time, and a 5 nm bandpass against 90% *v*/*v* DMF in water as the reference. For doxorubicin, the fluorescence emission spectra at an excitation wavelength of 470 nm were collected from 550 to 700 nm at an emission increment of 0.5 nm, a 0.05 s integration time, and a 5 nm excitation/emission bandpass against 90% *v*/*v* DMF in water as the reference. The calibration curves for gemcitabine HCl at 268 nm (Supplementary Material, Figure S2) and doxorubicin HCl at 595 nm emission/470 nm excitation (Supplementary Material, Figure S3) were used to calculate the loading of each drug (%wt.) in p-Gem/Dox.

### 3.5. In Vitro Cathepsin B-Catalyzed Cleavage

The cathepsin B-catalyzed cleavage of p-Gem/Dox was assessed in vitro for the release of the conjugated drugs. The conjugate (1.5 mg/mL) in cleavage buffer (pH 5.0; phosphate buffer containing 30 mM 1,4-dithiothreitol, 15 mM EDTA disodium salt dihydrate, 0.114 nM cathepsin B enzyme solution, 0.5% *v*/*v* Tween 20^®^, and 1% *v*/*v* DMSO) was incubated at 37 °C with continuous 360° rotation at 10 rpm using a fixed angle tube rotator (Thermo Fisher Scientific, Pittsburgh, PA, USA). A similar experiment without cathepsin B was set up as a control. Samples (200 μL) were withdrawn from the test and control at intervals (0–24 h), diluted with acetonitrile (400 μL) to precipitate the enzyme, and analyzed by reversed-phase HPLC (RP-HPLC).

### 3.6. In Vitro Hydrolytic Stability

The hydrolytic stability of p-Gem/Dox at 37 °C was evaluated in phosphate-buffered saline (PBS) (pH 7.4). A 0.37 mg/mL solution of p-Gem/Dox was prepared in PBS buffer to which Tween 20^®^ (0.5% *v*/*v*) and DMSO (1% *v*/*v*) were added and incubated at 37 °C with continuous 360° rotation at 10 rpm using a fixed-angle tube rotator for 24 h. At different time points, the samples were withdrawn for HPLC analysis as described in Section 3.5 above. The experiment was performed in triplicate.

### 3.7. In Vitro Cytotoxicity Evaluation in OVCAR-3 Cell Line

#### 3.7.1. Cell Culture

OVCAR-3 human OC cells were maintained in RPMI-1640 medium supplemented with 20% fetal bovine serum and 1% penicillin/streptomycin at 37 °C in a humid atmosphere with 5% carbon dioxide. The growth medium was replaced every 48 h.

#### 3.7.2. Cytotoxicity Evaluation of the Single Agents

The cytotoxicity of the single-drug PDCs, p-Dox, and p-Gem (Supplementary Material, Figure S4), against OVCAR-3 human OC cells was evaluated in vitro using CellTiter-Glo^®^ 2.0 assay (Promega, Madison, WI, USA). Cultured OVCAR-3 cells were seeded in 96-well plates at 10,000 cells/well (100 μL) and allowed to attach for 24 h. For direct comparison with the free drugs, the amount of p-Dox containing the same amount of doxorubicin HCl (DOX) in solution and the amount of p-Gem containing the same amount of gemcitabine HCl (GEM) in solution were used to treat the cells at different concentrations (0.005, 0.05, 0.5, 5, and 50 μg/mL). Cells that were treated with the culture media and DMSO in media were used as controls. Four replicates of each treatment were used. At 72 h post treatment, 100 μL of CellTiter-Glo^®^ 2.0 assay reagent was added to each well and mixed for 2 min on an orbital shaker to induce cell lysis. The plates were incubated in the dark at room temperature for 10 min, and luminescence was recorded using a Promega^TM^ GloMax^®^ plate reader (Promega, Madison, WI, USA). The percentage of viability was determined by normalizing the average luminescence of treated cells against untreated cells in media. The percentage of cell viability as a function of drug concentration was plotted and non-linear least-squares regression analysis was used to calculate the concentrations that inhibit the growth of 50% of the cells (IC_50_ values) using GraphPad Prism 10.1.1 (270) software [8].

#### 3.7.3. The Cytotoxicity Evaluation of the Combination Agents and Combination Index Analysis

The cytotoxic effects of the p-Dox + p-Gem admixture (50:50), GEM + DOX admixture (50:50), p-Gem/Dox, and GEM + DOX admixture (36.6:7) against OVCAR-3 cells were evaluated in vitro. As with the single agents, the amounts of the conjugates containing equivalent amounts of the free drugs in solution were used to treat the cells at concentrations 0.005, 0.05, 0.5, 5, and 50 μg/mL. Cells that were treated with the culture media and DMSO in media were used as controls. At 72 h post treatment, the viability assay was carried out using CellTiter-Glo^®^ 2.0 assay per the manufacturer’s protocol as described earlier. The cytotoxic effect of GEM + DOX interactions in OVCAR-3 cells was determined by calculating the combination index (CI) using the COMPUSYN software (version 1.0) based on the Chou–Talalay method [50].

## 4. Conclusions

The SPAAC-mediated step-growth polymerization method was used to prepare gemcitabine–doxorubicin combination PDC (p-Gem/Dox) with a high molecular weight, high drug loading, and a relatively narrow molecular weight distribution. Single PDCs containing gemcitabine and doxorubicin were prepared using the same method. The p-Gem/Dox had similar drug loading and cathepsin B-catalyzed drug release patterns as the single PDCs. The combination drug PDC was also more toxic to OC cells compared to the combination of individual PDCs of gemcitabine and doxorubicin. p-Gem/Dox holds the potential to reduce chemoresistance as a result of the delivery of combination therapeutics at high concentrations to the tumor. In addition, sequential passive–active tumor targeting facilitated by large molecular weight PDCs via the EPR effect and the use of GFLG as a selective cathepsin B substrate and mechanism of drug release, respectively, will improve therapeutic efficacy and eliminate non-specific toxicity to healthy cells. This approach has the potential to cure OC, which is typically diagnosed at an advanced stage.

## Figures and Tables

**Table 1:** The physical characteristics of the PDCs used in this study.

PDCs	M_w_ (kDa)	M_n_ (kDa)	PdI	M_p_ (kDa)	% Weight Doxorubicin	% Weight Gemcitabine
p-Gem/Dox	1360	1346.53	1.01	1240	7.00 ± 2.00	36.60 ± 0.24
p-Gem ^ø^	40.18	29.33	1.37	40.37	Not applicable	29.20 ± 0.30
p-Dox ^ø^	1800	1782.18	1.01	1920	10.30 ± 0.53	Not applicable

M_w_ = weight-average molecular weight; M_n_ = number-average molecular weight; PdI = polydispersity index = M_w_/M_n_; M_p_ = the molecular weight of the most abundant species based on the RI signal; and ^ø^ is from Ref. [24].

**Table 2:** The calculated IC_50_ values (mean ± SD) of the free drugs and the PDC equivalents in OVCAR-3 cells.

Drug Treatment	IC_50_ (µg/mL)
Single	
p-Dox	1.79 ± 0.17
Doxorubicin HCl (DOX)	0.17 ± 0.03
p-Gem	>50
Gemcitabine HCl (GEM)	>50
Combination	
p-Gem + p-Dox admixture (50:50)	3.87 ± 1.22
GEM + DOX admixture (50:50)	0.24 ± 0.12
p-Gem/Dox	0.99 ± 0.06
GEM + DOX admixture (36.6:7)	0.11 ± 0.04

## Data Availability

Data is contained within the article and Supplementary Material.

## Supplementary Materials

The supporting information can be downloaded at: https://www.mdpi.com/article/10.3390/ijms26062798/s1.
