# Supplementary material for: Gemcitabine–Doxorubicin Combination Polymer-Drug Conjugate Prepared by SPAAC Click Chemistry: In Vitro Characterization
Source: Int J Mol Sci. 2025 Mar 20;26(6):2798. doi: 10.3390/ijms26062798 (PMC11943159; doi:10.3390/ijms26062798)
Supplement: Supplementary file 1 [file ijms-26-02798-s001.zip › ijms-3505335-supplementary.pdf]

Supporting Information

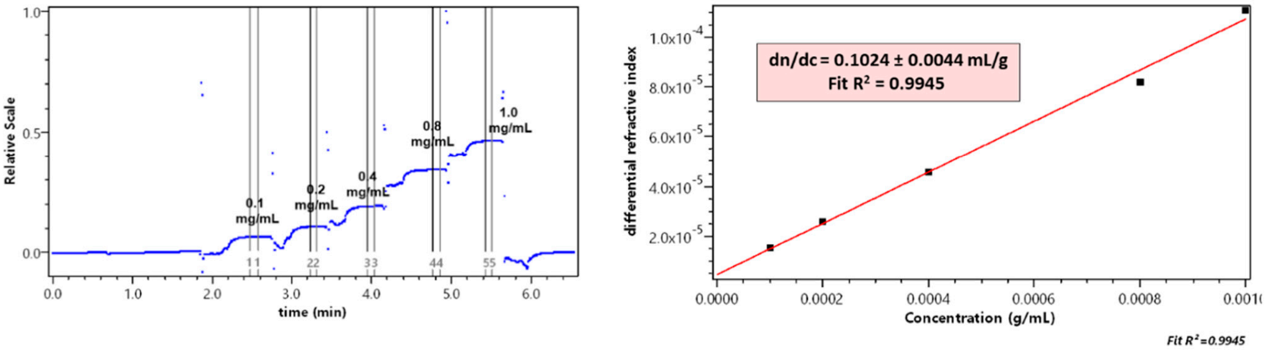

Figure S1. Left: Calibration curve for measuring the specific refractive index (dn/dc) of p-Gem/Dox; Right: Plot of the change in refractive index (dn) versus the change in concentration (dc) used for dn/dc determination of p-Gem/Dox.

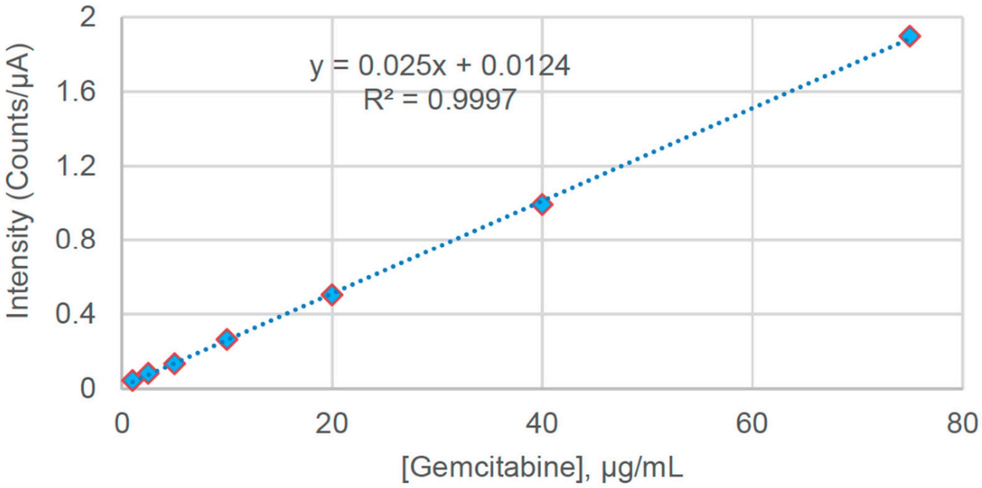

Figure S2. Calibration curve obtained from gemcitabine HCl solutions prepared with 90 % v/v DMF in water. UV-Vis absorbance intensity counts were measured at 268 nm.

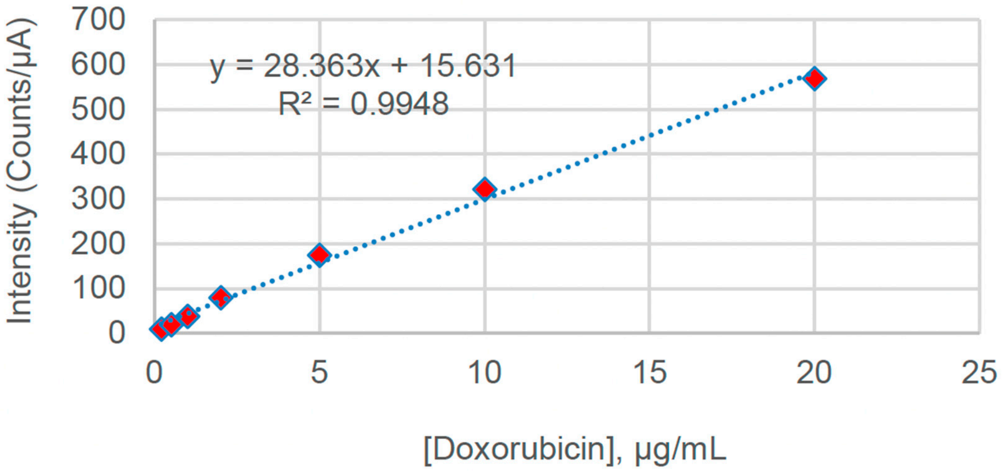

Figure S3. Calibration curve obtained from doxorubicin HCl solutions prepared with 90 % v/v DMF in water. Fluorescence emission intensity counts were measured at 595 nm (470 nm excitation).

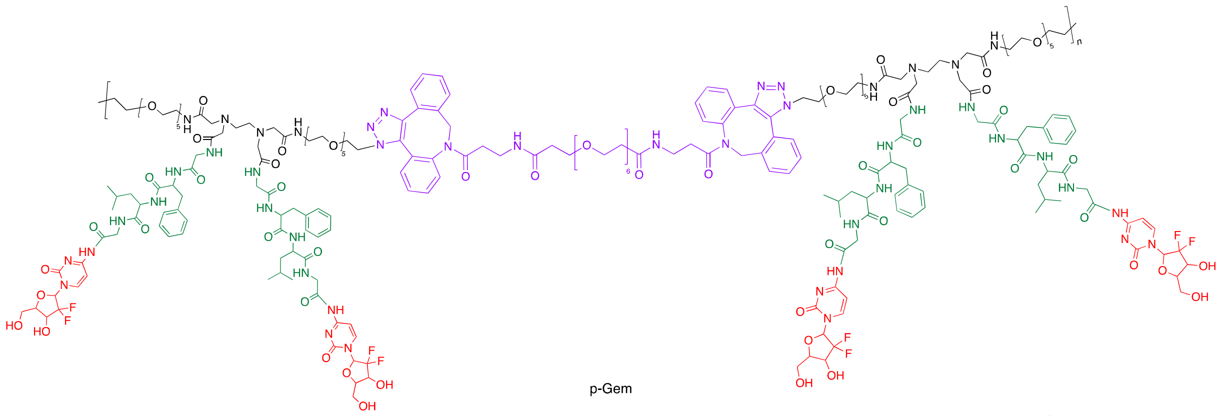

p-Gem

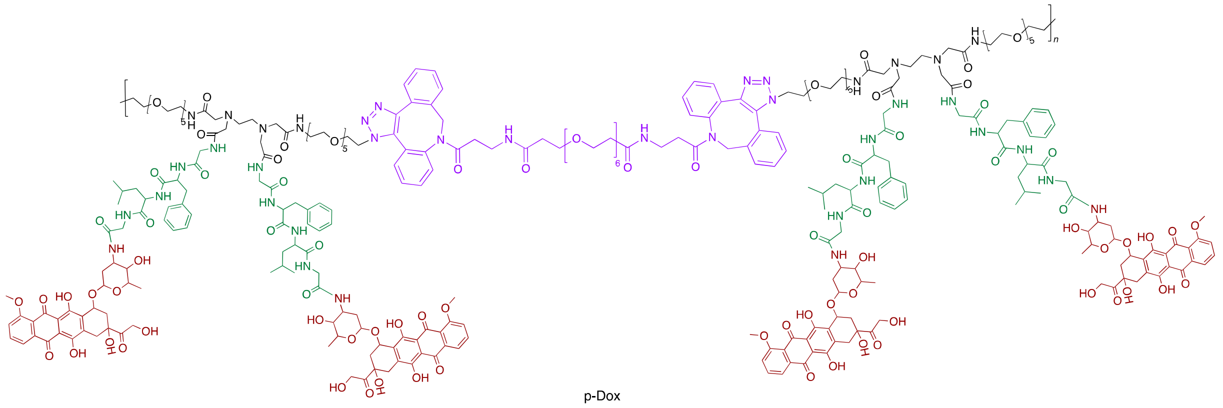

p-Dox

Figure S4. Top: Structure of gemcitabine PDC (p-Gem); Bottom: Structure of doxorubicin PDC (p-Dox).

p-Gem/Dox incubated with cathepsin B at pH 5.0 (test)

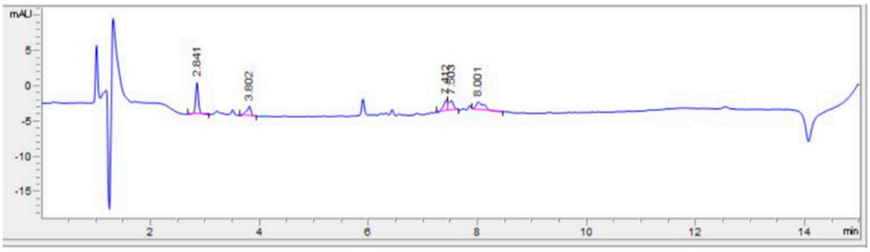

p-Gem/Dox incubated without cathepsin B at pH 5.0 (control)

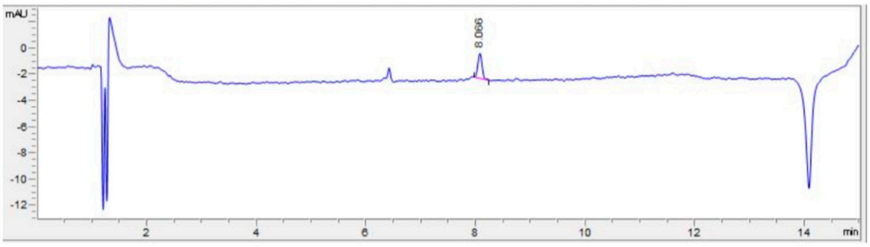

Figure S5. RP-HPLC chromatograms for p-Gem/Dox cleavage test solution (top) and control (bottom) at pH 5 showing the release of gemcitabine (peak retention time 2.8 min) and a doxorubicin fragment (peak retention time 3.8 min) from p-Gem/Dox (peak retention time 8.0 min) in the presence of cathepsin B. Peaks were monitored at 275nm.

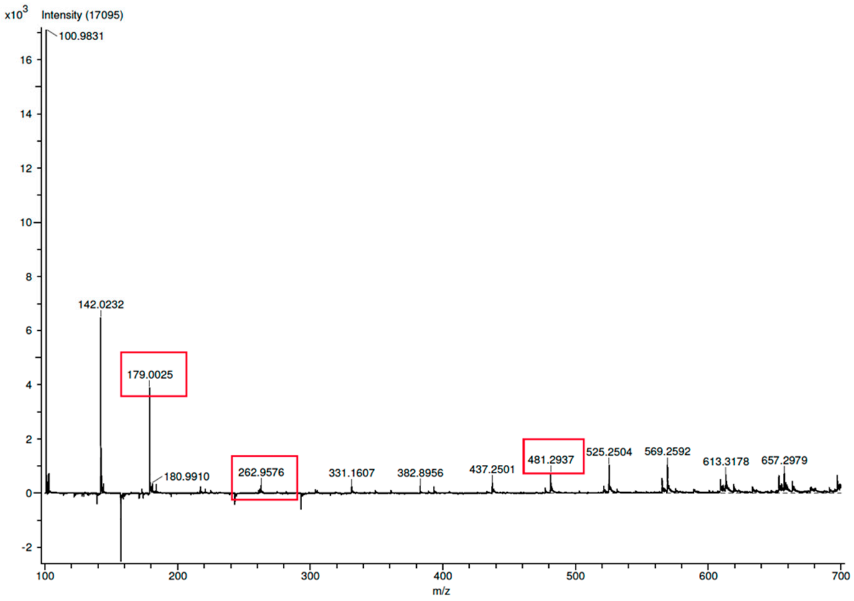

Figure S6. ESI-MS spectrum of p-Gem/Dox showing masses (m/z) corresponding to free gemcitabine (calculated 264.1 $[M + H]^+$, found 263.0), an adduct ion of the hydrophilic doxorubicin amino sugar moiety (calculated 179.0 $[M + CH_3OH + H]^+$, found 179.0), and a doxorubicin aglycone fragmentation product (calculated 482 $[M + H]^+$, found 481.3).
